# Efficacy of isoniazid prophylactic therapy in prevention of tuberculosis in children: a meta–analysis

**DOI:** 10.1186/1471-2334-14-91

**Published:** 2014-02-20

**Authors:** James Ayieko, Lisa Abuogi, Brett Simchowitz, Elizabeth A Bukusi, Allan H Smith, Arthur Reingold

**Affiliations:** 1 FACES (Family AIDS Care and Education Services), Centre for Microbiology Research, Kenya Medical Research Institute-Kisumu, Nairobi City, Kenya; 2 Department of Pediatrics, University of Colorado, Denver, CO, USA; 3 School of Medicine, University of California, San Francisco, USA; 4 School of Public Health, University of California, Berkeley, USA

**Keywords:** Tuberculosis, Isoniazid, Prophylaxis, Meta-analysis, Efficacy, Children

## Abstract

**Background:**

Children are highly susceptible to tuberculosis; thus, there is need for safe and effective preventive interventions. Our objective was to evaluate the efficacy of isoniazid in prevention of tuberculosis morbidity and mortality in children aged 15 years or younger by performing a meta-analysis of randomized controlled trials. To our knowledge, this is the first meta-analysis evaluating efficacy of isoniazid prophylaxis in prevention of tuberculosis in children.

**Methods:**

A systematic search of the literature was done to identify randomized controlled trials evaluating isoniazid prophylaxis efficacy among children. Each study was evaluated for relevance and validity for inclusion in the analysis. Subgroup analyses were conducted based on study quality, HIV status, tuberculosis endemicity, type of prophylaxis and age of participants.

**Results:**

Eight studies comprising 10,320 participants were included in this analysis. Upon combining data from all eight studies, isoniazid prophylaxis was found to be efficacious in preventing development of tuberculosis, with a pooled RR of 0.65 (95% CI 0.47, 0.89) *p* = 0.004 , with confidence intervals adjusted for heterogeneity. Among the sub-group analyses conducted, only age of the participants yielded dramatic differences in the summary estimate of efficacy, suggesting that age might be an effect modifier of the efficacy of isoniazid among children, with no effect realised in children initiating isoniazid at four months of age or earlier and an effect being present in older children. Excluding studies in which isoniazid was initiated at four months of age or earlier yielded an even stronger effect (RR = 0.41 (95% CI 0.31, 0.55) *p* <0.001). Data on the effect of isoniazid on all-cause mortality, excluding studies in which isoniazid was initiated in infants, yielded an imprecise estimate of mortality benefit (RR = 0.58 (95% CI 0.31, 1.09) *p* = 0.092).

**Conclusion:**

Isoniazid prophylaxis reduces the risk of developing tuberculosis by 59% among children aged 15 years or younger excluding children initiated during early infancy for primary prophylaxis (RR = 0.41, 95% CI 0.31, 0.55 p < 0.001) . However, further studies are needed to assess effects on mortality and to determine prophylaxis effectiveness in very young children and among HIV-infected children.

## Background

Tuberculosis (TB) is a potentially lethal, infectious disease caused by *Mycobacterium tuberculosis*[1]. Worldwide, there were an estimated 8.7 million incident cases of TB in 2011 and approximately 1.4 million deaths (430,000 among people who were HIV-infected) [2]. TB is now recognized as being second only to HIV/AIDS as the greatest killer worldwide caused by a single infectious agent and as the leading cause of mortality among people living with HIV [3], making it an important public health problem that requires effective intervention.

Children are highly susceptible to infection with *M. tuberculosis*, and once infected, are at much higher risk of progression to TB than adults [4]. This risk is greatest for infants and children < 2 years of age because of the immaturity of the immune system [5,6]. The situation is worse for those infected with HIV due to the accompanying immunosuppression that HIV infection causes. As a result, there is a need to protect this susceptible group from developing TB using safe and effective strategies.

Isoniazid (INH) is one of the first line medications used in multi-drug regimens for treatment of TB; as a single drug, it is also used to prevent *M. tuberculosis* infection and progression from latent infection to TB [7]. While many studies have demonstrated the effectiveness of INH prophylaxis in adults [8], there have been only a few studies in children, and these have produced conflicting results. Our objective was to evaluate the efficacy of isoniazid prophylaxis in prevention of tuberculosis morbidity and mortality in children ≤ 15 years of age by performing a meta-analysis of RCTs (randomized controlled trials). This paper attempts to estimate more precisely the efficacy of INH prophylaxis in all children by pooling data from existing studies. To our knowledge, this is the first meta-analysis to explore this question, other than a Cochrane review published in 2009 that was limited to a single study of HIV-infected children [9].

## Methods

We included only randomized controlled trials (RCTs) involving children aged ≤ 15 years, regardless of their HIV status, comparing INH (4–20 mg/kg) with placebo or no prophylaxis. The studies had to have been published in English in peer-reviewed journals. Publications were of any date through October 2012. Studies were restricted to RCTs to provide the strongest evidence for the efficacy of INH.

### Search methods and data collection

**Figure 1:**
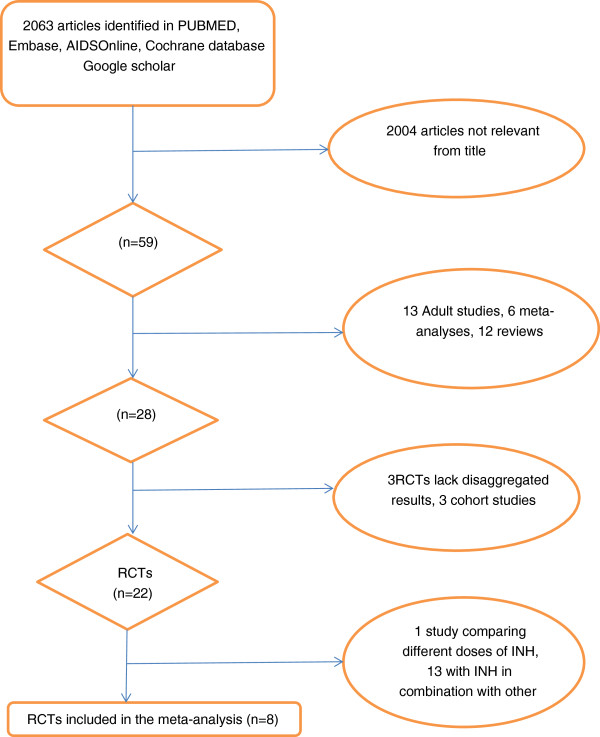
Diagram of systematic search (Flow Diagram).

RCTs were identified through a search of PubMed, Embase, Aidsonline, Google Scholar and Cochrane database electronically and by reviewing the references of identified articles (Figure 1). The following search terms were used: isoniazid, INH, prophylaxis, preventive therapy, children, tuberculosis, TB, HIV as listed in titles and abstracts, and randomized controlled trial as publication type. The search included all English articles through October 2012.

Two reviewers searched and evaluated trials for inclusion in the analysis, and data were collated and checked by both reviewers before inclusion. Discrepancies were resolved by consensus.

The quality of each RCT included in this analysis was graded by use of a validated score that included the following items (10): randomization of participants, double-blind evaluation, and a description of participants who withdrew from or dropped out of the trial. The scoring gives one point for each item present. If randomization is concealed and the method of double-blind evaluation is appropriate, the study is assigned one additional point for each of these, yielding a score of 0–5 points. Studies that scored three or more points were considered to be high quality studies, while those with a score below three were considered to be low quality studies. To supplement this score we used the Cochrane criteria to grade concealment of treatment allocation as: adequate (A), unclear (B), inadequate (C), or allocation was not used (D) [10].

### Statistical analysis

Most studies presented their results using risk ratios (RRs). For those that included both adults and children, we disaggregated the raw data and calculated RRs and the corresponding 95% confidence intervals for the children separately. Analysis was performed using the intention-to-treat principle.

The summary RR estimates were calculated using both the fixed effects inverse variance weighting method [11] and the DerSimonian-Laird random effects method [12]. Heterogeneity among studies was assessed using the general variance-based method [13]. If heterogeneity was present, confidence intervals in the fixed effects model were adjusted to account for between-study variance using the method presented by Shore et al. [14].The advantage of this approach is that it still weights by precision, while also taking heterogeneity into account.

Heterogeneity of INH efficacy between studies was investigated using the I^2^ statistic. An I^2^ value of >50% was considered an indicator of substantial heterogeneity (values of I^2^ equal to 25%, 50%, and 75% were deemed to represent low, moderate, and high heterogeneity, respectively). In addition, significant heterogeneity was deemed to be present when the Chi^2^ test statistic was greater than the degrees of freedom. To explore heterogeneity among studies, we performed subgroup analyses based on the HIV status of the children, duration of administration of INH prophylaxis (< or ≥ six months), age of participants (arbitrary cut-off set at 5 years of age), primary and secondary prophylaxis, TB endemicity where the study was conducted, and quality of the study. Primary prophylaxis here refers to administration of INH for prevention of TB infection while secondary prophylaxis refers to administration of INH to prevent TB disease. Sensitivity analyses were also carried out to assess the influence of individual studies on the summary effect estimate.

Publication bias was assessed by plotting the logarithm of the RR for each study by its standard error (SE) [15] and explored using a funnel plot. Publication bias was also assessed using the Egger [16] and Begg tests [17]. An absence of publication bias would be indicated by an even dispersion of effect sizes around the pooled effect estimate. All meta-analyses risk estimates, graphs and plots were performed using STATA, version 12.1.

## Results

Of 2063 articles initially identified, eight studies (comprising 10,320 children) were included in this analysis (Figure 1) [18-24], two of which were presented in a single article by Madhi et al. [19]. Madhi’s article [19] included two independent sets of participants; those who were HIV positive and those who were HIV negative but had been exposed to HIV (i.e. born to a HIV- infected mother but, themselves HIV uninfected). We considered HIV infection as a potential effect modifier and chose to treat these two subsets as separate studies.

Table 1 provides information about the characteristics of the trials included in the analysis. Among the eight studies, two included participants (*n* = 810) who were HIV-infected [18,19]; one included HIV negative participants born to HIV-infected mothers (*n* = 806) [19], and the other five included only HIV negative participants (*n* = 8,703) irrespective of their exposure status [20-24]. Three of the eight studies included participants who were tuberculin skin test (TST) positive only [21,22,24], and two studies excluded participants who had BCG scars [22,24]. Three of the trials were conducted in southern Africa, one in India, one in France, one in Kenya, one in the US, and one at multiple sites in Mexico, the US and Canada. The quality of six of the trials [18-21,23] was rated as high; the remaining two trials were rated as low quality [22,24].

**Table 1:** Studies included in the pooled analysis

**Author(Year)**	**Sample size(n)**	**Age of participants**	**Dosage**	**Location**	**Quality score**	**Risk Ratio**	%Weight using FEM*
Madhi et al. (2011) [19]	547	<4 months	10–20 mg/kg	South Africa and Botswana	5A	0.98(0.7,1.39)	32.4%
Madhi et al. (2011) [19]	806	<4 months	10–20 mg/kg	South Africa and Botswana	5A	0.86(0.57,1.29)	22.8%
Zar et al. (2007) [18]	263	<51 months	10(8–12)mg/kg	South Africa	5A	0.28(0.1,0.78)	3.6%
Gupta et al. (1993) [24]	167	5–15 years	15 mg/kg	India	1D	0.61(0.3,1.25)	7.5%
Debre et al. (1973) [22]	2,307	5–14 years	5–15 mg/kg	France	1D	0.40(0.15,1.05)	4.0%
Egsmose et al. (1965) [20]	406	0–15 years	5–10 mg/kg	Kenya	5A	0.38(0.16,0.88)	5.2%
Comstock et al. (1962) [23]	3,074	2 months–15 years	4–8 mg/kg	Alaska	5A	0.33(0.07,1.64)	1.5%
Mount et al. (1961) [25]	2,750	0–15 years	4–6 mg/kg	USA, Canada, Mexico	5A	0.40(0.27,0.61)	22.9%
**Total**	**10,320**						100%

*FEM-Fixed Effects Model.

In two of the trials, INH prophylaxis was administered for a duration of less than six months, while in the other six it was administered for six months or longer. The duration of follow-up among the studies varied from 5.7 months to 10 years. Six studies indicate that they used tablets, the studies by Gupta et.al and Debre et.al did not indicate the formulation used. Two of the eight studies used INH for prevention of primary infection; three studies used it for secondary prevention of TB, and the remaining three for both primary and secondary prevention. All studies with the exception of the study by Comstock et al., followed up participants actively. The studies used a combination of clinical signs and symptoms, radiological findings and bacteriological investigations to make a diagnosis of TB. All studies, with the exception of that by Gupta et al., give a comprehensive explanation of how the diagnosis was arrived at.

The results of the meta-analysis are shown in Table 2. Combining data from all eight studies, there was a 35% reduction in the risk of developing TB among those who were randomized to receive INH (RR = 0.65, 95% CI 0.47, 0.89 *p* = 0.004). In subgroup analyses, only the age of participants was found to yield substantial differences in the summary estimate of the effect of INH, suggesting that age might be an effect modifier of the efficacy of INH prophylaxis among children. No effect was noted in children on whom INH was initiated at an age of four months or earlier for primary prophylaxis of TB (RR =0.93, 95% CI 0.71, 1.21 *p* = 0.29) while INH had an effect in older children aged 5 to 15 years (RR = 0.53, 95% CI 0.30, 0.94 *p* = 0.014). Excluding studies focused on INH prophylaxis in infants (Madhi et al.) yielded a greater protective effect (RR = 0.41 (95% CI 0.31, 0.55) *p* <0.001).

**Table 2:** Summary of pooled risk ratios and subgroup analyses of INH efficacy

**Groups**	**Number of studies**	**Number of participants**	Fixed effects risk ratio(95% CI*)	**Random effects risk ratio(95% CI)**	**Heterogeneity Chi **^**2**^**( *p *)**
All studies	8	10,320	0.65(0.47,0.89)^#^	0.53(0.38,0.80)	18.56(0.00968)
High quality	6	7,845	0.66(0.45,0.98)	0.55(0.35,0.87)	17.55(0.00357)
Low quality	2	2,474	0.53(0.30,0.94)	NA	0.47(0.49303)
HIV negative	6	9,509	0.55(0.40,0.75)	0.52(0.36,0.75)	8.57(0.1277)
HIV positive	2	810	0.86(0.41,1.81)	0.58(0.17,1.94)	5.14(0.02335)
TB endemic	5	2,189	0.78(0.55,1.11)	0.67(0.45,0.99)	8.93(0.06281)
TB non endemic	3	8,130	0.40(0.27,0.57)	NA	0.05(0.9736)
Primary TB prophylaxis	2	1,353	0.93(0.71,1.21)	NA	0.23(0.63119)
Secondary TB prophylaxis	3	5,224	0.44(0.31,0.61)	NA	1.05(0.59104)
Excluding studies INH was initiated at age ≤4 months	6	8,966	0.41(0.31,0.55)	NA	1.84(0.87086)
High quality excluding studies INH was initiated at age ≤4 months	4	6,492	0.38(0.27.0.53)	NA	0.43(0.93385)
INH initiated at age ≤4 months	2	1,353	0.93(0.71,1.21)	NA	0.23(0.29)
Age <5 years	3	1,616	0.86(0.57,1.30)	0.77(0.48,1.23)	5.14(0.07644)
Age 5–15 years	2	2,474	0.53(0.30,0.94)	NA	0.47(0.49303)
INH for ≥6 months	6	7,845	0.66(0.45,0.98)	0.55(0.35,0.87)	17.55(0.00357)
INH for <6 months	2	2,474	0.53(0.30,0.94)	NA	0.47(0.49303)

*Adjusted for heterogeneity when appropriate.

^#^1 minus the risk ratio gives the protective effect, e.g. for the first row, 0.65 corresponds to 35% protection.

**Figure 2:**
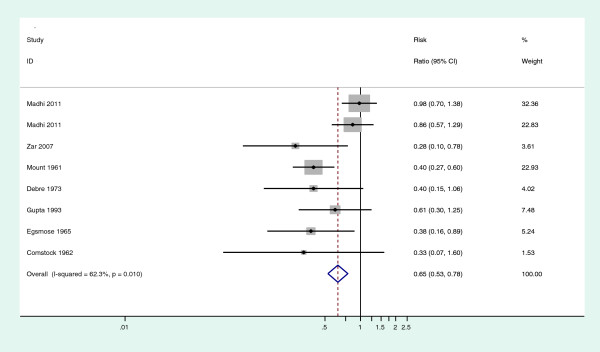
Forest plot of all the eight studies included in the meta-analysis.

On pooling the eight studies, marked heterogeneity in the efficacy of INH was observed (Chi^2^ 18.56, df = 7, I^2^ = 64.3% *p* = 0.01) (Figure 2); upon conducting a sensitivity analysis, we noted that by excluding the two studies by Madhi et al. that included infants on whom INH had been initiated at four months of age or earlier, the heterogeneity disappeared (I^2^ = 0.0%, p = 0.799), demonstrating that these two studies were the principal contributors of the heterogeneity observed in the meta-analysis.

Only four trials (comprising 2,391 children) provided data on all-cause mortality and their results were inconclusive (mortality RR = 0.86, 95% CI 0.63, 1.17, *p* = 0.168) (Table 3), (I^2^ = 12.2%, *p* = 0.332). Excluding studies in which INH was initiated in infants at an age of four months or earlier yielded an imprecise estimate of 42% reduction in mortality (RR = 0.58 (95% CI 0.31, 1.09) *p* = 0.092).

**Table 3:** Effects of Isoniazid prophylaxis on all cause mortality

**Study**	**HIV status of participants**	**Participant ages**	**Risk ratio(95% CI)**	***p value***
Madhi et al. (2011) [19]	Positive	<4 months(median 96 days, range 91–120 days)	1.00(0.68,1.48)	0.986
Madhi et al. (2011) [19]	Negative	<4 months(median 96 days, range 91–120 days)	0.90(0.49,1.65)	0.733
Zar et al. (2007) [18]	Positive	<51 months (median 24.7 months, IQR 9.4–51.6 months)	0.46(0.22,0.95)	0.015
Egsmose [20]	Negative	0–15 years	0.94(0.33,2.66)	0.91
*All four studies combined*			0.86(0.63,1.17)	0.168
*Excluding studies that INH was initiated at age***≤***4 months*			0.58(0.31, 1.09)	0.092

Of the eight trials, all but one reported on the adverse effects of INH prophylaxis. The most serious adverse effects reported were deaths of two children following overdoses of INH in the study by Comstock et al. [23]. In the same study, only 0.8% of the participants had adverse effects that necessitated discontinuation of INH. In the study by Debre et al., 1% of the participants had adverse effects that necessitated discontinuation of INH. The adverse effects reported by the other studies were less serious and did not warrant complete discontinuation of INH.

**Figure 3:**
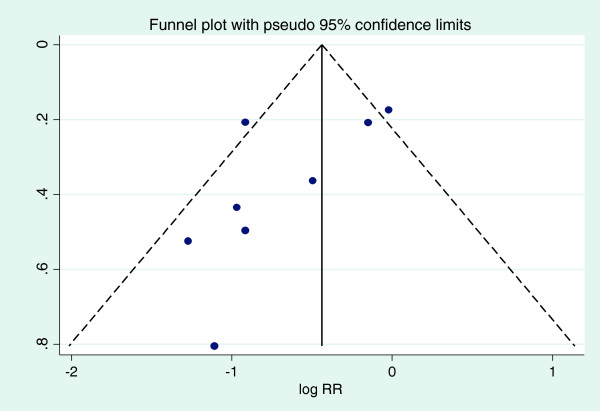
Funnel plot of all eight studies included in this meta-analysis.

With regard to assessment of publication bias, Figure 3 presents the funnel plot of all the studies included in this meta-analysis (Egger test *p* = 0.050, Begg test *p* = 0.099.), upon excluding the two infant studies by Madhi et al. the asymmetry disappeared, (Egger test *p* = 0.598, Begg test *p* = 0.26).

## Discussion

This meta-analysis brings together trials done in varied settings. The children in the various trials were of diverse ages and varying HIV status. Upon pooling the studies, INH was found to provide substantial protection against TB among children aged ≤15 years (RR = 0.65, 95% CI 0.47, 0.89 p = 0.004), however, there was marked heterogeneity in the efficacy of INH (Chi ^2^ 18.56, df = 7, I^2^ = 62.3% *p* = 0.01). We conducted a sensitivity analysis to explore the observed heterogeneity. Most of the heterogeneity was noted to be due to the two studies by Madhi et al.(also referred to as the infant studies in this article) [19], primarily due to two reasons. First, the results of the two studies by Madhi et al. were notably different from the results of the other studies, in that these two studies yielded point estimates much closer to the null compared to the results of the other studies, with confidence intervals that included the null, while the rest of the studies had point estimates and confidence intervals that showed that INH conferred a protective effect. Second, both the random effects and the fixed effects models gave these two studies more weight because of their relatively large size. One plausible explanation for the null results in the Madhi studies may be due to overdiagnosis of TB given that only few of the cases were confirmed microbiologically and many cases only met minimal clinical criteria [19]. After excluding the two studies by Madhi et al., the heterogeneity disappeared (I^2^ = 0.0%, *p* = 0.799) and our analysis yielded an even greater effect estimate (RR = 0.41 (95% CI 0.31, 0.55) *p* <0.001).

One possible explanation for the discrepancy between findings from Madhi et al. and from the remaining studies has to do with the age of the participants at initiation of INH. Whereas subjects in the Madhi et al. trials were infants (median age 96 days, range 91–120 days) at the time of INH initiation, the remaining studies involved mostly older children, suggesting that age may modify the effect of INH prophylaxis on the development of TB. This hypothesis is supported by subgroup analyses based on five studies that investigated differential INH efficacy by age. INH was found to be more effective among children aged 5–15 years (RR = 0.53, 95% CI 0.30, 0.94 *p* = 0.014) than among children less than five years of age (RR = 0.85, 95% CI 0.54, 1.35 *p* = 0.25) (Table 2). As mentioned previously, INH did not appear to confer any protective effect against TB in the infant studies (RR = 0.93, 95% CI 0.71, 1.21 *p* = 0.29).The lack of effect observed may be due to the relative immaturity of the innate immune system among younger as opposed to older children considering existing evidence suggesting that the dosage delivered in these studies was sufficient to achieved therapeutic levels [26]. We also acknowledge that age might be a confounding factor for prior TB exposure, given that in the Madhi et al. study, INH was used for primary prophylaxis compared to the other studies in which it was used for either secondary prophylaxis or both primary and secondary prophylaxis. INH is known to be effective for the preventive treatment of latent TB which would be more prevalent among older children but it might not be as effective in preventing TB infection. We therefore cannot fully rule out the possibility that age may be a surrogate marker for TB infection thus explaining the higher efficacy seen in older children consistent with the results of the primary versus secondary prophylaxis subgroup analysis. While age may, in fact, be an effect modifier of the efficacy of INH, additional alternative explanations proposed by Madhi et al. for the null results in their studies include INH resistance, poor compliance, and the specificity of the study end points [19]. Other differences in the studies by Madhi et al. compared to the rest were that the population had no known history of TB exposure and the participant ages were more uniform compared to the other studies.

Minimal differences in the efficacy of INH prophylaxis were seen in the subgroup analyses based on duration of administration of INH and study quality. Six trials administered INH for a duration of six months or longer and showed a 34% reduction in the risk of developing TB among those randomized to receive INH, RR = 0.66 (95% CI 0.45, 0.98) *p* = 0.02; the rest of the trials administered INH for less than six months and still demonstrated a protective effect against TB, RR = 0.53 (95% CI 0.30, 0.94) *p* = 0.014. These results suggest that administration of INH for less than six months may be as effective as longer courses of prophylaxis among non-HIV infected children. This finding, which is consistent with that of other studies demonstrating only a small advantage of a 12-month over a 6-month course of INH preventive therapy [27], may have important public health implications. This would be useful if applied in high TB endemic areas where the duration and intensity of exposure might be a critical factor affecting effectiveness of INH prophylaxis. While current guidelines for isoniazid preventive therapy recommend six months of prophylaxis [18], an equally effective but shorter course could result in improved adherence to therapy in children without HIV [28]. While the studies in this meta-analysis that administered a shorter course of prophylaxis were of generally low quality (Table 1) and should therefore be interpreted with caution, these findings justify further research into the optimal duration of INH preventive therapy in children, which remains uncertain [27].

Subgroup analyses based on HIV infection status found a strong protective effect of INH against TB among HIV-negative children (RR = 0.55, 95% CI 0.40, 0.75 *p* = 0.001), but no evidence of an effect among HIV-positive children (RR = 0.86, 95% CI 0.41, 1.81 *p* = 0.187). However, it is worth noting that the analysis of HIV-infected children was limited by the inclusion of only two studies; one in which INH was used for primary prophylaxis in HIV-exposed infants (Madhi et al.) and the other for secondary prophylaxis in older HIV-infected children (Zar et al.). We note, however that the Zar et al. study found INH to be highly effective and is further discussed below. As mentioned above, age may, itself, modify INH efficacy, complicating interpretation of these results. Therefore, we are unable to make a conclusive statement concerning INH efficacy among HIV-infected children and urge further research into this question. To our knowledge, the two studies we included in this subgroup analysis are currently the only studies on the efficacy of INH in prevention of TB that have been conducted in HIV-infected children.

The results also suggest that the efficacy of INH was greater in TB non-endemic regions (RR = 0.40, 95% CI 0.27, 0.57 *p* < 0.001) compared to TB endemic regions (RR = 0.78, 95% CI 0.55, 1.11 *p* = 0.08). One plausible explanation as to why isoniazid may have been less efficacious in endemic regions is reinfection of the children with a different strain of *Mycobacterium tuberculosis*. However, it is important to note that the results of this subgroup analysis may be misleading because the studies in TB endemic regions contained a large proportion of very young children compared to studies in the TB non-endemic regions, and the studies that contained this category of participants (Madhi et al.) yielded null results; this finding, combined with the fact that the studies by Madhi et al. received more weight in both the fixed effects and the random effects models, may explain the null result in this subgroup analysis. Upon excluding these two studies, we obtained a summary estimate of RR 0.44(0.27, 0.72) p < 0.001, suggesting that INH confers a protective effect in TB endemic areas as well.

Four of the eight studies included in this meta-analysis reported results on all-cause mortality. Combining the results of these studies (comprising 2,391 children) showed little evidence of an effect of INH on all-cause mortality RR = 0.86 (95% CI 0.63, 1.17) *p* = 0.168, consistent with the findings of studies conducted on adults [29]. This estimate however may be misleading , because two of the four studies included were the infant studies described above, where no protective effect against TB or mortality benefit had been demonstrated; these two studies received 77.8% of the weight in this subgroup analysis. We thus excluded these two studies to get a more reliable estimate (RR = 0.58 (95% CI 0.31, 1.09) *p* = 0.092); this estimate, though imprecise, may suggest a mortality benefit of INH prophylaxis. We are limited in our ability to draw firm conclusions on mortality benefits of INH prophylaxis due to the limited data available, and thus recommend further studies.

A legitimate concern with expanded use of INH prophylaxis among children is the potential for serious adverse events resulting from therapy. This meta-analysis, however, suggests that INH is relatively safe in children. The most serious adverse events reported in the studies included were the deaths of two children following overdoses of INH in the study by Comstock et al. [23]. In the same study, however, only 0.8% of the participants experienced adverse events that necessitated discontinuation of the medication. The adverse events reported in the other studies were generally mild and did not warrant discontinuation of INH. The study by Zar et al. reported that the safety and tolerability of INH prophylaxis in children were excellent, even among the HIV-infected subgroup receiving highly active antiretroviral therapy. Widespread use of INH over several years has shown that a dosage of 5 mg/kg of body weight is unlikely to cause serious toxic effects [23], a conclusion substantiated by this meta-analysis and the results of other INH prophylaxis trials [25,30-32]. Another concern is INH resistance; previous reviews have not found a statistically significant elevated risk of isoniazid-resistant TB among individuals previously treated with isoniazid preventive therapy [33].

The study by Zar et al. demonstrated a marked protective effect in terms of prevention of TB (RR 0.28 (95% CI 0.1, 0.78) *p* = 0.005) and TB mortality (RR 0.46 (95% CI 0.22, 0.95) *p* = 0.015) among HIV-infected children and is therefore worth commenting on [18]. The study by Zar et al. was a landmark study that was conducted on HIV- infected children in a TB endemic region and demonstrated marked benefits of INH in terms of reducing morbidity as well as mortality and was instrumental in informing policy on INH prophylaxis among HIV-infected children [34]. We note that most of the differences in mortality and incidence of TB were observed within two months after randomization, presenting the possibility that some of the children selected to participate in the study already had subclinical TB and the INH was actually treating (as monotherapy) rather than preventing TB. This scenario, if true, may have led to an overestimation of the efficacy of INH as preventive therapy. However, it is worth noting that if the aforementioned concern is true, the findings by Zar et. al present valuable evidence demonstrating that INH is useful in reducing TB and mortality in HIV-infected children with advanced disease (WHO stage 3 or 4), regardless of whether a diagnosis of TB is made or not. Diagnosis of TB in this category of children presents a challenge, especially in resource limited settings [34].

The risk of bias in this meta-analysis arises mostly from inclusion of two studies that were of generally low quality. The study by Debre et al. was not conducted in a blinded fashion, while the study by Gupta et al. did not give details of how the randomized controlled trial was conducted; the authors acknowledged irregularities and defaults in drug intake in the latter study which may have influenced their results [22,24]. Concerning all of the studies, generally, diagnosis of TB is very difficult in children [34], particularly in infants and this may have led to failure to make a diagnosis, misdiagnosis or over diagnosis of the outcome, leading to a biased estimate of the efficacy of INH preventive therapy. This scenario is highly likely due to the diverse diagnostic criteria used in the different studies which is a major weakness in this analysis. Misdiagnosis is equally likely in the treatment and control groups so the effect was likely to be non-differential and therefore bias estimates towards the null.

Publication bias has the potential to bias the results of a meta-analysis [9]. We used a funnel plot to look for evidence of publication bias [35]. While there was some suggestion of asymmetry in the funnel plot, we are not convinced that publication bias is a serious threat to our findings. However, it is important to note that other factors, apart from publication bias, can cause asymmetry in a funnel plot and affect the outcome of the Egger and Begg tests; in addition, the validity and interpretation of the Egger and Begg tests have been debated [36]. It should also to be noted that the sensitivity of both the Egger and Begg tests is low when the analysis contains fewer than ten studies, as was the case with our meta-analysis [37]. To establish whether publication bias was a concern, we estimated the pooled RR from the logarithm of the RR corresponding to the largest studies, which was approximately −0.4; we exponentiated this value, yielding an estimate of RR = 0.67, close to the estimate of our pooled summary measure (RR 0.65) showing that in this case, publication bias may not be influential in altering the overall summary estimate. In addition, because the studies by Madhi et al. were different from the other studies because of the ages of the participants at INH initiation and were responsible for the asymmetry on the funnel plot, we excluded them and re-did a funnel plot; after exclusion of these two studies there was no asymmetry (Egger test *p* = 0.598, Begg test *p* = 0.26), providing no evidence of publication bias.

Limitations associated with this meta-analysis include the fact that only eight studies were deemed suitable for inclusion, compromising the statistical power of the subgroup analyses. Diverse TB diagnostic criteria were used in the different studies which is a major weakness in this analysis due to lack of standardization across studies. Attempts have been made to standardize TB case definition in children for research purposes to prevent this scenario in the future [38]. In addition, the studies were of a mixed nature with some evaluating efficacy of INH in primary TB prevention while others evaluating secondary TB prophylaxis yet others evaluating efficacy of preventing both primary and secondary TB, this limits comparability across the studies. Another limitation was that we restricted our search to English language articles and thus may have missed studies published in other languages. Furthermore, we included only RCTs in this meta-analysis, which poses the challenge of generalizability because trial participants are different from the general population, owing to the fact that trials often have strict eligibility criteria and those who agree to participate may be very different from the general population.

## Conclusion

The results of our analysis suggest that INH prophylaxis reduces the risk of TB by 59% among children ≤ 15 years of age, excluding a subset of young children on whom INH was initiated at four months of age or earlier for primary prophylaxis (RR = 0.41, 95% CI 0.31, 0.55 p < 0.001). INH confers a protective effect against TB among HIV negative children; however, we had insufficient data to make a definitive conclusion on efficacy of INH in preventing TB among HIV-infected children. In contrast to the WHO recommendation to give all HIV- infected children over 12 months of age isoniazid preventive therapy, our results show no effect of isoniazid preventive therapy among this group (though from limited data) and justifies more investigation. The results further suggest that INH is not effective among the youngest children and that there is little evidence of a mortality benefit in children of any age. Based on our results, we recommend the administration of INH to children at risk, especially those with smear positive contacts, because, as the evidence indicates, INH will reduce their risk of developing TB and thus help lower TB disease burden and TB-related years lost to disability (YLD).

Due to the fact that there are limited data on the efficacy of INH in children ≤ five years of age, in HIV-infected children, and on overall mortality, we recommend that further studies be carried out to answer conclusively the question of whether INH is effective among very young children and children who are HIV-infected, as well as to determine the optimum duration of INH preventive therapy and to assess the mortality benefit among children.

## Abbreviations

AIDS: Acquired Immunodeficiency Syndrome; BCG: Bacille Calmette-Guerin (vaccine); HIV: Human Immunodeficiency Virus; INH: Isoniazid; RCT: Randomized controlled trial; TB: Tuberculosis; TST: Tuberculin skin test; WHO: World Health Organization.

## Competing interests

The authors declare that they have no competing interests.

## Authors’ contributions

Concept development and study design: JA, AR. Identification of studies: JA, LA. Supervision of the study: AR Data interpretation and revision of manuscripts for important intellectual content: JA, LA, BS, EB, AS, AR. All authors read and approved the final draft for publication.

## Authors’ information

James Ayieko: M.B.Ch.B, MPH; Lisa Abuogi: MD, MPH; Brett Simchowitz: MPH, Medical Student; Elizabeth A Bukusi: M.B.Ch.B, MMed, MPH, Ph.D Allan H Smith:MD, MPH, Ph.D: Arthur Reingold: MD.

## Pre-publication history

The pre-publication history for this paper can be accessed here:

http://www.biomedcentral.com/1471-2334/14/91/prepub
